# Characterization by surface plasmon resonance of electrochemical biosensors developed with organophosphorous hydrolase enzymes applied to detection of neurotoxin

**DOI:** 10.1186/1753-6561-8-S4-P98

**Published:** 2014-10-01

**Authors:** Diego Custódio De Souza, Antonio Carlos Seabra

**Affiliations:** 1 Universidade de São Paulo, São Paulo, Brazil

## 

The biosensor devices were first researched by Clark and Lyons in 1962, aiming to measure the blood glucose level. Currently, there is a popularization of blood glucose meters and various technologies have been developed mainly for applications in medical diagnostics and environmental monitoring. However, there is a wide range of applications for biosensors not yet available. The immobilization of bioactive species that interact directly with the analyte, called bioactive surface, is often considered the most difficult step in the biosensors development for new applications. The objective of this work is to demonstrate the characterization of the bioactive surface developed by self-assembled monolayers technique using the electrostatic adsorption principle. For this, we used three different concentrations of solutions composed of covalent bonds between carbon nanotubes and three different biomaterials, Polyethyleneimine, Deoxyribonucleic Acid and Organophosphorous Hydrolase enzymes. These enzymes catalyze the hydrolysis of phosphorus compounds such as organophosphorous neurotoxins. The different polyelectrolyte solutions were characterized by the technique of surface plasmon resonance, and simultaneously, self-assembled monolayers were formed on the surface of a glassy carbon electrode. The enzymes activity was analyzed by cyclic voltammetry for the different concentrations of the solutions used. The results showed that the surface plasmon resonance characterization was interesting to promote the control of electrostatic adsorption. The variation of the surface plasmon resonance angle corresponds the intensity and saturation of electrostatic interactions between the layers. Biosensors developed with solutions of higher concentration, consequently, widened the detection limits, and increased the resolution of the electrical signal due to the lower saturation level, and higher slope signal. In applications to measure low concentrations of analyte, adequate control of the concentrations of polyelectrolytes solutions reduces costs in the development of biosensors especially, the cost of isolated enzymes.
